# The Diabetic Lung: Insights into Pulmonary Changes in Children and Adolescents with Type 1 Diabetes

**DOI:** 10.3390/metabo11020069

**Published:** 2021-01-26

**Authors:** Chiara Mameli, Michele Ghezzi, Alessandra Mari, Giulia Cammi, Maddalena Macedoni, Francesca Chiara Redaelli, Valeria Calcaterra, Gianvincenzo Zuccotti, Enza D’Auria

**Affiliations:** 1 Department of Pediatrics, V. Buzzi Children’s Hospital, 20154 Milan, Italy; Michele.ghezzi@asst-fbf-sacco.it (M.G.); alessandra.mari@unimi.it (A.M.); Giulia.cammi@unimi.it (G.C.); maddalena.macedoni@asst-fbf-sacco.it (M.M.); francesca.redaelli@asst-fbf-sacco.it (F.C.R.); valeria.calcaterra@asst-fbf-sacco.it (V.C.); gianvincenzo.zuccotti@unimi.it (G.Z.); enza.dauria@unimi.it (E.D.); 2 Department of Biomedical and Clinical Science “L. Sacco”, Università di Milano, 20157 Milan, Italy; 3 Allergology and Pneumology Unit, V. Buzzi Children’s Hospital, 20154 Milan, Italy; 4 Pediatric and Adolescent Unit, Department of Internal Medicine, University of Pavia, 27100 Pavia, Italy

**Keywords:** type 1 diabetes, children, diabetic lung injury, pulmonary function, diabetes-related complications

## Abstract

Historically, the lung was not listed and recognized as a major target organ of diabetic injury. The first evidence of diabetic lung involvement was published fifty years ago, with a study conducted in a population of young adults affected by type 1 diabetes (T1D). In recent years, there has been mounting evidence showing that the lung is a target organ of diabetic injury since the beginning of the disease—at the pediatric age. The deeply branched vascularization of the lungs and the abundance of connective tissue, indeed, make them vulnerable to the effects of hyperglycemia, in a way similar to other organs affected by microvascular complications. In this review, we focus on pulmonary function impairment in children and adolescents affected by T1D. We also cover controversial aspects regarding available studies and future perspectives in this field.

## 1. Introduction

Type 1 diabetes (T1D) is a chronic disease characterized by immune-mediated destruction of pancreatic beta-cells, which leads to partial, or more often, absolute insulin deficiency [1]. Currently T1D is the most common form of diabetes in childhood. It was estimated that 85% of all cases worldwide are diagnosed in individuals under 20 years of age [2].

The incidence of T1D is increasing globally in recent decades [3,4,5,6,7,8,9,10]. A EURODIAB study showed that in 22 European countries, the T1D incidence rate in children under 14 years old increased by 3.4% yearly, from 1989 to 2013 [11]. A similar trend was reported in the United States, describing an increase in incidence of T1D of 1.9% per year, between 2002 and 2015, in children under 20 years of age [12].

The burden of T1D is largely attributed to the development of both macrovascular and microvascular complications, which result in an increase in morbidity and mortality during the patient’s lifespan, even after the introduction of intensive insulin therapy and screening programs [13,14]. Life-expectancy for individuals with younger-onset disease is on average 16 years shorter, compared to people without diabetes, and 10 years shorter for those diagnosed at an older age, due to diabetes-related complications [15].

Kidneys, heart, blood vessels, nerves, and eyes are considered the major target organs for diabetes-related injuries and a large volume of literature is published in this field [16]. However, considering that T1D is a systemic disease, it is likely that it could induce a variety of deficits in many other organs and systems.

The first evidence of diabetic lung involvement was published fifty years ago with a study conducted in a population of young adults affected by T1D [17]. Since then, an increasing number of research studies were published, mainly focused on the adult population and especially those affected by type 2 diabetes [18,19].

In recent years, there is mounting evidence showing that the lung is a target organ of diabetic injury since the beginning of the disease, in pediatric age [20,21]. The deeply branched vascularization of the lungs and the abundance of connective tissue, indeed, make them vulnerable to the effects of hyperglycemia in a manner similar to other organs affected by microvascular complications.

In this narrative review, we focus on pulmonary function impairment in children and adolescents affected by type 1 diabetes. We also address controversial aspects regarding available studies and future perspectives in this field.

## 2. Methods

This narrative review aims to focus on the impairment of lung function in children and adolescents with T1D. Each author identified and critically reviewed the most relevant published studies in English (original papers and reviews). The following keywords were used to search for papers published up to October 2020, in each author’s field of expertise—lung damage, pulmonary function, diabetes type 1, asthma, children, and adolescents. The following electronic databases were searched—PubMed, Scopus, EMBASE, and Web of Science. The contributions were collected, and the resulting draft was discussed among authors to provide a theoretical point of view, which is considered an important educational tool in continuing medical education. The final version was then recirculated and approved by all co-authors.

## 3. Pathogenesis of Diabetes-Induced Lung Damage

Historically, the lung is not listed and recognized as a major target organ of diabetic injury. As with other less investigated disease-related impairments (e.g., gustatory and olfactory alteration) [22], pulmonary damage is mostly subclinical and difficult to detect [18].

The biochemical pathogenesis of diabetes-related lung damage is yet to be fully understood and more research is necessary to establish a strong biological basis.

**Figure 1:**
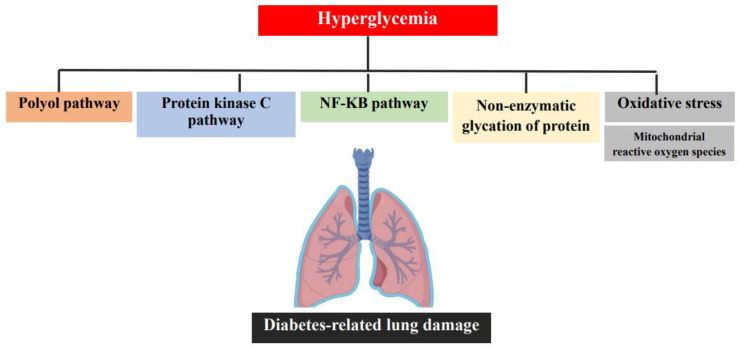
Biochemical mechanisms and signaling pathways potentially involved in diabetes-related lung damage.

As in other complications, long-lasting hyperglycemia seems to be the leading factor in promoting injury. A variety of pathways, including oxidative stress, non-enzymatic glycation of proteins, NF-κB pathway, and activation of the protein kinase C, polyol pathway, and sorbitol production, are upregulated as a consequence of chronic exposure to hyperglycemia. Therefore, the damage is the result of a multitude of complex and inter-related pathways (Figure 1). Unfortunately, the exact contribution of each of these is currently unknown [23].

The mechanisms of lung damage are thought to include chronic inflammation, microangiopathy of lung vasculature, and autonomic neuropathy. All these factors contribute to ultrastructural abnormalities in endothelial and epithelial cells, interstitium and vascular wall, and also lead to respiratory muscular dysfunction [24,25,26,27,28].

The lungs of patients with diabetes were found to be infiltrated by inflammatory cells such as macrophages, neutrophils, and lymphocytes [29]. This infiltration, combined with the accumulation of collagen, increased fibrotic tissue, and interstitial hyperplasia leads to alveolar compression and a reduction of the alveolar space. Autoptic studies also showed an increased width of the alveolar wall in diabetic patients (versus controls), due to a thickening of both the alveolar epithelium and alveolar capillary basal lamina [27,28].

The expression of pro-inflammatory and pro-fibrotic factors, such as TNF alfa PAI-1 ND and the connective tissue growth factor seems to play a central role in the development of pulmonary fibrosis [29].

Chronic hyperglycemia also promotes an increased synthesis of reactive oxygen species (ROS), which are involved in triggering the onset of diabetic microangiopathy, both in the lungs and in other tissues. ROS activate the nuclear enzyme poly(ADP-ribose) polymerase (PARP), which is considered to be one of the most important effectors of oxidative–nitrosative injury while inhibiting GADPH, with the consequent activation of four different pathways, all of which are responsible for damaging lung tissue in diabetic patients [23].

The mechanisms sustained by GADPH inhibition are the polyol pathway, the formation of advanced glycation end products (AGEs), the activation of protein kinase C (PKC) with a subsequent increase of NFkB and the hexosamine pathway flux [23]. The polyol pathway is also a source of ROS production. The nuclear factor kB (NFkB) upregulates the expression of genes that encode for proteins such as pro-inflammatory cytokines, adhesion molecule, cyclooxygenase-2, and nitric oxide synthase (iNOS) [23]. PKC dysfunction seen in diabetic patients’ lungs has many effects on the vascular system, such as altered permeability, increased collagen production, blood flow abnormalities, inflammation, and cell apoptosis [23,29,30,31,32]. Mitochondrial dysfunction and redox imbalance were also reported to be potentially pathogenic in the lungs of patients affected by diabetes. Impaired function in NAD+ dependent enzymes, such as NQO1, DLDH, and sirt3 were described in rat models [33]. Nonetheless, the role of mitochondria in diabetic lung injury, a major source of ROS and oxidative stress, is yet to be fully understood [33].

The role of autonomic neuropathy in lung injury is less clear but not completely excluded [34]. The vagus nerve, involved in parasympathetic innervation of the heart, also has a role in the innervation of the respiratory muscles. In this context, autonomic neuropathy might result in impaired nervous control in the lung tissue and in the respiratory muscles, and it could also alter the regulation of pulmonary blood flow. Some authors showed how cardiovascular autonomic neuropathy can be associated with reduced pulmonary functions in adolescents with T1D. However, more studies are needed to support these data [34].

## 4. Pulmonary Function in Children and Adolescents with T1D

In the literature, several studies (both cross-sectional and longitudinal) among adults with T1D show how diabetes is associated with subclinical abnormalities affecting both lung function and gas transfer with reduced carbon monoxide diffusion capacity—albeit with some conflicting results. In particular, it seems that adults with diabetes exhibit a high risk of restrictive impairment of lung function with significant decreases in many parameters, including a lower vital capacity, medium expiratory flow, expiratory residual volume, total lung capacity, forced vital capacity, and forced expiratory volume in one second, when compared to healthy subjects [35].

In recent years, a number of authors investigated pulmonary function in children and adolescents affected by T1D. Studies in literature analyzing pulmonary function and outcomes in children with diabetes are few and results are less compelling than those in adults, yielding conflicting conclusions (Table 1). In fact, a discrepancy in the type of defect affecting lung function emerged from these studies—some reported only reduced forced vital capacity (FVC), together with normal forced expiratory volume in 1 s (FEV1) [25,36], while others reported a reduction of both FVC and FEV1 [37], and yet others showed normal lung volumes, but increased airway resistance [38,39].

Cazzato et al., with a cross-sectional study, analyzed the presence of pulmonary impairment in children affected by T1D, plus any correlations with diabetic factors and complications [40]. No patient had a history of clinical or radiological evidence of pulmonary disease.

Thirty-eight children with diabetes (10 ± 1.8 years), with a mean duration of disease of 3.2–3.3 years at the time of the analysis, and 41 healthy age-matched subjects, underwent pulmonary function studies, through the analysis of flow-volume curves with spirometry and whole-body plethysmography. A total of 34% of the children with diabetes were studied at the onset of the disease.

Significant differences were found between the patients and the controls for all function indices, with the exception of expiratory reserve volume (ERV) and total lung capacity (TLC). The adjusted values of FVC, FEV1, and RV were found to be significantly lower in children with diabetes than in healthy ones (*p* = 0.0001, *p* = 0.01, *p* = 0.003 respectively) and these differences were also found in the subgroup at disease onset, with the exception of RV (*p* = 0.003, *p* = 0.04 respectively).

The functional pattern described by this study was consistent with decreased lung elastic recoil, caused by prolonged hyperglycemia resulting in excessive non-enzymatic glycosylation of connective tissue [27,46,47], and with diminished elasticity. This is because FVC was impaired and the RV/TLC ratio was higher than in controls (*p* = 0.0002), even at the onset of disease (*p* = 0.04), without evidence of reduced FEV1/FVC or TLC.

In this study, the authors did not find a significant relationship between the pulmonary function test and microalbuminuria or mean HbA1c concentrations. Furthermore, there was no correlation between duration of disease and respiratory function variables or with age at onset of diabetes mellitus.

On the contrary, they highlighted how the differences in pulmonary function found in diabetic patients were present also in the subgroup at the onset of disease, similar to what Suresh et al. later reported—in their study, pulmonary function abnormalities appeared within 3 years of diabetes diagnosis in 51.2% of children with T1D [48].

In a study conducted by Mohamad et al., among 60 children with T1D (mean age 10.5 ± 2.32 years; disease duration 2.45 ± 0.6 years), and 50 healthy controls who underwent spirometry, a significant reduction in all spirometric parameters was highlighted in T1D children.

In contrast to the results of Cazzato et al., children with poor glycemic control (HbA1c > 8%) were found to have significant impairment in lung functions, as compared to those of good glycemic control [42].

Again, Scaramuzza et al. found reduced lung volumes in 42 T1D children compared to 30 healthy controls [20], while in the populations of the other studies, increased airway resistance was the only abnormality in lung function. Van Gent et al. did not report any reduction of FVC or FEV1 among 27 T1D children, comparing the results to reference values and not to a healthy control group [39]. The thickening of the basal membrane, described in adult patients with type 1 diabetes, might explain the increased airway resistance.

A recent study by Anik et al. showed an increase in airway resistance by oscillometry, while spirometric values that revealed a peripheral airway impairment demonstrated by the reduced zFEF 75 and zFEF 25–75; those findings were associated with disease severity and duration [45].

Recently, Martin-Frias et al. conducted a case-control study in a population of 100 children with diabetes with a disease duration of 6.2 ± 3.8 years (average age 13 years, all non-smokers and without chronic complications, lung disease or other comorbidities), compared to 77 healthy controls. Spirometry and plethysmography were performed, and the relationship with duration of diabetes, degree of metabolic control, and presence of diabetic complications were analyzed and then corrected for pubertal stages.

The authors found that patients with diabetes showed non-significant lower FVC and FEV1, and higher FEV1/FVC ratio. After correction for pubertal stage, the only parameter to reach significance was the FEV1/FVC ratio, which was considerably higher in patients with diabetes than in controls. After correction for pubertal stage, TLC and RV were found to be notably higher, while airway resistance was markedly lower in children with diabetes, when compared to healthy controls. Furthermore, children with diabetes with poor metabolic control (HbA1c > 7.5%) presented a lower FEV1/FVC ratio when compared to children with good metabolic control, but no differences were found in the other parameters.

Furthermore, no differences were found in the pulmonary function in relation to disease duration, even though the duration of the disease was <10 years in 82% of the children [21]. This finding (Martin-Frias et al.) suggests a subclinical involvement of lung parenchyma, as shown by the increase in the FEV1/FVC ratio and the trend towards lower values of FVC and FEV1, which, according to the authors, might be related to the effects of diabetes on pulmonary mechanics and elasticity.

Martin-Frias et al. also underlined how pubertal stage impacted on changes in pulmonary function. The only other study that took into consideration the pubertal stage while evaluating pulmonary function was the one conducted by Cazzato and Bernardi [40], in which pubertal stage did not affect results.

The Martin-Frias findings were in line with previous reports by Buckingham et al., who described an FVC reduction in 19% of patients (which included, adults, smokers, and patients with pulmonary diseases) [36], as well as previous reports by Primhak et al. [37]. FVC reduction was also reported by Al Saadi et al., who evaluated lung function in poorly controlled T1D Saudi children and adolescents [43], but not by other authors, such as Villa [41].

Similarly, the reduction of FEV1 initially reported by Cazzato and Villa [46], was not confirmed by later studies conducted by Verrotti, Van Gelt, Al Saadi, and Villa himself [39,41,43].

Moreover, no reduction in FEV1 or FVC was described by Pieniawska et al. in Polish children and adolescents with type 1 diabetes [44]; whereas the author confirmed an increase in the FEV1/FVC ratio and showed a decrease in peak expiratory flow (PEF), when compared to the normal values. Furthermore, no relationship was found between disease duration, HbA1c percentage, and spirometric parameters, apart from vital capacity, which seems to be significantly reduced in diabetic patients with poor metabolic control (HbA1c > 7%).

### 4.1. Pulmonary Diffusing Capacity

The lungs are primarily involved in gas exchange, mostly oxygen and carbon dioxide, through the alveolar capillary membrane.

Lung diffusing capacity for carbon monoxide (DLCO) is a measure of gas conductance across the alveolar tissue membrane into capillary erythrocytes, and subsequent chemical binding to hemoglobin. DLCO is influenced by the alveolar-capillary membrane conductance and pulmonary capillary blood volume.

Evidence in literature suggests an impaired DLCO in adults with T1D, due to both, a reduced conductance of the alveolar-capillary membrane and a reduced pulmonary capillary blood volume [49]. Recently, lung diffusion capacity was investigated during exercise. Wheatley et al. demonstrated that adults with D1T at peak exercise present a decreased DLCO when corrected for cardiac output (DLCO/Q), and the decrease in diffusing capacity was associated with a reduction in oxygen saturation [50]. Additionally, they found a decreased membrane diffusing capacity for carbon monoxide (DMCO) when corrected for cardiac output (DMCO/Q). It was suggested that the limitation in gas transfer becomes increasingly functionally significant, the more the transit time of red blood cells through the lung was shortened [50].

In the literature, there are few studies investigating gas exchange and lung diffusing capacity for carbon monoxide in children affected by T1D, and it seems that impaired DLCO could be related to a significant impairment in pulmonary capillary blood volume [20].

To our knowledge, the first author to highlight an impaired DLCO in children with diabetes was Cazzato [40]; previous reports in pediatric age, in fact, described a normal DLCO in this group of patients, when compared to the reference values [39].

In their cross-sectional study, Cazzato et al. compared DLCO in thirty-eight children with diabetes (without clinical or radiological pulmonary disease) and in forty-one healthy children. Sixteen patients had reduced DLCO (42%). Adjusted values of this index were significantly lower than in controls (*p* = 0.001) and this difference emerged also in the group of patients at the disease onset stage (*p* = 0.001). Reduction in DLCO was neither correlated with duration of disease nor with other diabetic complications. The only significant relationship found was between reduced DLCO and female diabetic patients (*p* = 0.03), suggesting sex as a predisposing factor for pulmonary complications.

The presence of this alteration at the beginning of the disease, according to the authors, might be related to the early process of collagen glycosylation, which is stabilized and stopped when diabetes mellitus is diagnosed and insulin therapy is promptly begun. With the stabilization of glycemic levels and a decrease in glycosylation, pulmonary function might also be stabilized, thus, explaining the lack of correlation with disease duration.

In 1999, Weynand described a thickened alveolar epithelial and pulmonary capillary basal lamina in human subjects with type 1 diabetes [27]. This finding is considered to be the initial lesion in the development of diabetic microangiopathy.

Subsequent studies [20,41] confirmed a reduced diffusing capacity of carbon monoxide in children with diabetes early in childhood, and in patients with poor metabolic control.

Villa et al. studied the association of alveolar capillary diffusion with disease-related variables in thirty-nine children with type-1 diabetes and thirty healthy children. Authors found that children with poor metabolic control (HbA1c > 8%) had lower values of DLCO corrected for alveolar volume (DLCO/VA) than diabetic children with good glycemic control and healthy control children (*p* < 0.05), and that the predicted DLCO/VA percentages statistically correlated with HbA1c levels (*p* = 0.013).

An alternative explanation authors gave to the diminished DLCO/VA observed in children with T1D was that children might have low DLCO levels because high oxygen binding lowers CO binding, as the binding curve of glycosylated hemoglobin might be left-shifted. This biochemical mechanism would also plausibly explain why patients who had the highest HbA1c had the lowest DLCO.

Later, Scaramuzza et al. retrospectively evaluated DLCO and its components—membrane diffusing capacity (DM) and pulmonary capillary blood volume (Vc), in forty-two children with T1D and thirty healthy age- and sex-matched controls. Their findings confirmed a significant reduction in DLCO when compared to the controls (*p* = 0.0001), but they also found a difference in DLCO components. When differentiating DM and Vc compartments, a significant impairment was seen only in Vc rather than DM (respectively *p* = 0.0001 and 0.798) [20]. This finding was previously reported in adult populations [25] as an early sign of pulmonary microangiopathy involvement. To our knowledge, the Scaramuzza et al. study highlighted for the first time in literature, the difference in DLCO compartments in children with diabetes. In this study, similar to Cazzato’s findings, the authors did not find any correlation with HbA1c levels, nor were correlations found between the pulmonary diffusion capacity and nephropathy or retinopathy. A slight correlation was found only with the subclinical signs of autonomic neuropathy, in contrast to what was observed by other authors [51].

Martin-Frias et al. found higher DLCO values in patients with T1D than in the controls. However, after correction for pubertal stage, DLCO and VA were found to be lower and DLCO/VA was higher in T1D patients than in controls, although these differences did not reach statistical significance [21]. Their results supported the hypothesis of a subclinical alteration of pulmonary diffusion capacity in children with type 1 diabetes mellitus, even though DLCO was not influenced by metabolic control or disease duration.

The study conducted by Lee et al., involving T1D adolescents and adults, found that their resting and peak exercise pulmonary carbon monoxide diffusing capacity was not significantly different to that of healthy controls. However, they reported that people with longer duration of disease (type 1 diabetes diagnosis >10 years) presented lower DLCO than controls, and this difference was more evident during high-intensity exercise. None of the participants displayed evidence of microangiopathy, and there was no significant relationship between the DLCO and HbA1c levels. These findings suggest that diabetes-specific impairment of pulmonary diffusion is a progressive process [52].

## 5. T1D and Asthma

Asthma and type 1 diabetes mellitus are two chronic immune-mediated diseases of primary relevance during childhood. Incidence of type 1 diabetes increased in the last few decades, in parallel to that of childhood asthma [53,54].

Asthma is the most frequent chronic disease, accounting for 9.3% of children in the United States [55]. It is a heterogeneous disease, characterized by chronic inflammation with different phenotypes and endotypes; the most frequently observed type in children underpins a T helper type-2 (Th-2) inflammation, a pathway also associated with the pathogenesis and development of other allergic diseases [56].

Immune dysregulation in type 1 diabetes, on the contrary, seems to be linked to T helper-1 (Th-1) cells [56] and it appears to be associated with a higher prevalence of other autoimmune coexisting conditions [57].

This leads to the hypothesis of a counter-regulation of Th-1 and Th-2 cells—subjects with type 1 diabetes should have a lower risk of developing Th-2 related allergic diseases (like asthma). This concept encapsulates the so-called “Th-1/Th-2 paradigm”—a reduction of Th-1 compartments should lead to a Th-2 expansion and vice versa [56,58].

In particular, the Th-2 associated pathway is characterized by the expression of IL4, IL13, IL-5, and IL-9; IL 5 and IL13 seem to inhibit Th-1 responses and Th-1-associated interferon gamma, while IL-2 seems to downregulate Th-2 responses. It is hypothesized that a different genetic background might be involved in the predisposition to asthma, atopic dermatitis, and T1D [59], even though shared candidate genetic markers are also implicated in T1D and atopic eczema (albeit inconclusively).

In recent decades, the Th-1/Th-2 paradigm evoked new interest and gave rise to a number of studies analyzing the relationship between asthma or atopic diseases and type 1 diabetes mellitus, with discordant results (Table 2).

Sheikh et al. compared skin prick tests in children with diabetes and healthy subjects and found no difference in positivity rate, suggesting that factors other than the Th1/Th2 balance were implicated in the reduced pulmonary manifestation [69].

Additionally, Tosca et al. failed to demonstrate a lower prevalence of sensitization to allergens in children and adolescents with diabetes [60]. The authors concluded that other T-cell subsets might be involved in the pathogenesis of autoimmune and allergic disorders and that the Th1/Th2 paradigm might be an oversimplification.

A recent study conducted in Austria showed an allergen sensitization rate that was not significantly different between T1DM children and healthy controls (*p* = 0.625). Furthermore, a comparable number of patients in both groups reported allergic symptoms (*p* = 0.43) [61].

Similarly, a Finnish study showed that allergic sensitization was just as frequent in children with T1D, as in controls. Mean total IgE levels and the prevalence of high IgE levels (100 kU/L) did not differ between the two groups either [62].

The meta-analysis by Cardwell et al., considering 25 studies from Europe and North America, reported a lower risk for atopic diseases, rhinitis, and asthma in children with type 1 diabetes, as compared to healthy populations (*p* < 0.05) [70]; this was later confirmed by Cakir’s findings [71].

Recent studies showed how children with diabetes and asthma show a unique cytokine expression, different from that of solely diabetic, solely asthmatic, or healthy children—in particular, a dysregulation of IL-10 (a defect in the regulation of IL-10 secretion) and higher serum levels of both IL-12 and IL-18 [72,73].

The hygiene hypothesis argues that early environmental stimulation by infections is necessary to achieve a mature and balanced repertoire of immune responses [74] and it was proposed to explain the parallel increase of T1D and atopic diseases, due to reduced stimulation of the immune system through early infections [75].

On the contrary, Black et al. demonstrated a higher rate of asthma in children and adolescents with diabetes, finding a prevalence of asthma of 10% in children with type 1 diabetes. They further highlighted a significant correlation between asthma and poor glycemic control in children with diabetes, as compared to children with only diabetes [63].

Klamt et al. and other authors found similar results to Black et al. in their European studies, i.e., a higher prevalence of IgE-mediated allergies and asthma in children and adolescents with diabetes, as compared to healthy controls [64,76]. Similarly, a higher prevalence of asthma in children with D1T compared to controls (with poor glycemic control influencing asthma risk) was found in a Taiwanese study by Hsiao et al. and in the Brazilian cohort of Villa-Nova [65,66].

These findings could be explained by the coexistence of Th1 and Th2 dysregulation, with a higher incidence of asthma in Th1-mediated diseases, hypothesizing a common environmental pathway [77].

Recently, Hortenuber et al. analyzed the prevalence of asthma and its influence on glycemic control in patients with diabetes under 20 years of age, listed in the German and Austrian registry. The study involved 51,926 patients with type 1 diabetes, 3.4% of whom were diagnosed with asthma or prescribed asthma-specific drugs, corresponding to a similar prevalence compared to the general population of healthy Germans [78]. Furthermore, patients with asthma who received higher insulin doses (*p* < 0.01), had decreased height-standard deviation scores and increased body mass indices (*p* < 0.01 and 0.04 respectively) and experienced more severe hypoglycemia (*p* < 0.01). HbA1c did not show any difference between subjects with and without asthma overall. The higher therapeutic demand could be explained by stress, less physical activity leading to an increase in insulin-resistance, asthma-associated inflammation and use of drugs, in particular steroids [67].

Asthma prevalence analogous to the control population confirms the results previously found by Tosca et al. in 2009—authors described no differences in asthma episodes during the participants’ lifespan (“lifetime asthma”) between subjects with type 1 diabetes mellitus and the control groups, even when analyzed by considering sensitization or non-sensitization towards common allergens. Among patients with diabetes and asthma, respiratory disease was defined as “mild intermittent”, indicating that type 1 diabetes does not seem to downregulate allergic sensitization, but seems to reduce the severity of clinical expression [60].

Recently Metsälä et al. studied a cohort of children and adolescents born in Finland between 1981 and 2009 and evaluated the relationship between asthma and type 1 diabetes, using a multistate modelling approach. After adjusting for sex and birth decade, they found that previous diagnosis of asthma increased the risk of subsequent type 1 diabetes by 41%, whereas previous diagnosis of type 1 diabetes decreased the risk of subsequent asthma by 18%. These findings were not explained by age at diagnosis, birth decade, sex, maternal asthma/diabetes or birth-related factors, and implied that the association between the diseases was more complex than previously thought, and its direction might depend on the sequential appearance of the diseases [68].

## 6. Controversial Aspects and Future Directions

Overall, the available studies highlight some critical issues that compromise the strength of the conclusions. Here, we discuss the most interesting aspects and suggest some ideas for future directions.

### 6.1. Research Methodology

- The available literature was scarce compared to publications on other diabetes-related complications or subclinical damage in pediatric age.
- The applied research design was not always appropriate. Even though most studies had a control group, 4 were designed without including healthy children and adolescents as controls. Failure to use a control group makes it difficult to draw meaningful conclusions.
- Characteristics of the study population. The age range of the T1D population recruited varied widely between the studies published so far. In particular, the upper age range varied the most, with young adults (up to 22 years of age) also included in pediatric studies. Pubertal stage was not taken into consideration by the majority of the authors. Only 2 research groups adjusted their results according to the pubertal stage.
- Studies at disease onset.
- Currently, only one study explored the pulmonary function at T1D onset. The majority of authors recruited patients some years after disease onset. We believe that including patients at the very beginning of diabetes onset would enable a better understanding of the progression of lung damage during the pediatric age. Prospective studies would surely add new insights into the subclinical progression of lung involvement.

### 6.2. Disease-Related Variables and Therapy

#### 6.2.1. Indicators of Glycemic Control

In the available studies, glycated hemoglobin was the only parameter used to define the metabolic control of T1D patients. This is not surprising, considering that these studies were performed between the 80s’ and the early 2000s’. The advent of technology, including continuous glucose monitoring (CGM), deeply changed clinical practice in recent years. CGM technologies gave healthcare professionals unprecedented access to a range of new indicators of glycemic control. Among these, time in range (TIR) shows promising results. In fact, new evidence suggests that TIR can predict the risk of long-term diabetes complications [79]. Given its recent introduction, no data are currently available on the relationship between TIR and lung involvement. Therefore, the introduction of TIR as endpoint in future clinical trials would be of interest.

#### 6.2.2. Therapy

Most studies were conducted before or at the advent of modern continuous subcutaneous insulin therapy. The availability in 2020 of the Hybrid Closed Loop System would give researchers the opportunity to design new studies that include patients wearing this new device.

In conclusion, there is a need to increase the number of well-designed case-control studies, with large cohorts of pediatric patients (up to 18 years old). Moreover, long-term prospective studies including patients at disease onset and considering the most recent markers of metabolic control would help us to describe the natural evolution of lung damage.

## 7. Conclusions

In reviewing the currently available studies of lung function in children and adolescents affected by T1D, we encountered data that are consistent with what is already described in adults and in vitro. The data available in the literature support the idea of a subclinical involvement of the lung as a target organ of diabetes-related complications, in particular affecting pulmonary mechanics and elastic recoil, as well as lung diffusion capacity. This impairment seems to be present from a young age, perhaps since diagnosis. Different studies reported conflicting results about whether this damage might be related to duration of disease, glycemic control, or other disease-related factors.

Poor lung function at the transitional age could be a risk factor for developing early onset chronic obstructive pulmonary disease, as shown by evidence from other conditions with more longitudinal data [80].

For this reason, although there is currently not enough evidence to support the introduction of routine screening of lung function in children and adolescents affected by T1D, as not enough longitudinal studies were carried out in the pediatric and transitional age, and most of them were limited to a small sample size, we believe that it might be useful to further characterize lung involvement in T1D with longitudinal studies from childhood to adult ages, with a larger numbers of patients and a longer history of disease. A clearer picture of how diabetes affects lung volumes, flows, and gas exchange could help in creating an appropriate screening program and in choosing the most useful lung function test; on the other hand, it would also provide useful information for the implementation of inhaled insulin therapy.

Moreover, we also evaluated the relationship between asthma and type 1 diabetes mellitus—two increasingly frequent chronic immune-mediated diseases of primary relevance during childhood. Many studies were carried out on whether there might be a connection between asthma or atopic diseases and type 1 diabetes mellitus, with controversial results demonstrating that the Th1/Th2 paradigm might be an oversimplification.

## Figures and Tables

**Table 1:** Studies on pulmonary function in children and adolescents with T1D.

	Study Design	No. of T1D Patients (age, ys)	Spirometry	Plethysmography	DLCO	Other Findings	Correlation with Study Variables
Buckingham et al. 1986 [36]	Observational without control group	375 (7–25 ys)	<FVC	-	-	-	No significant relationship between decreased vital capacities and duration of diabetes
Primhak et al. 1987 [37]	Observational, Case-control	88 (6–17 ys)	<FVC	-	-	-	No correlation between reduced FVC and duration of disease or mean HbA1c
Verrotti et al. 1993 [38]	Observational, without control group	68 (6–22 ys)	Normal	Normal (>Raw)	-	Subgroup analysis: T1D with microalbuminuria vs. T1D without microalbuminuria	Microalbuminuria is not a risk factor for the development of abnormal pulmonary function.
Van Gent et al. 2002 [39]	Observational, without control group	27 (age range NA, mean 12.8 ± 5 ys)	Normal	Normal (>Raw)	Normal	-	No correlation with age, disease duration or HbA1c
Cazzato et al. 2004 [40]	Observational, Case-control. 38% of cases were studied at onset of disease.	38 (1–12 ys)	<FVC, <FEV1	<RV >RV/TLC	<	Results corrected for pubertal stage All the alterations except < RV were also found in subgroup at onset of disease	<TLCO in female gender No correlation between pulmonary function indices and mean HbA1c, microalbuminuria, duration of disease
Villa et al. 2004 [41]	Observational, case-control	39 (5–14 ys)	Normal	-	<DLCO/VA	Subgroup analysis: patient with good vs. poor metabolic control (Hba1c < 8% vs. ≥8%)	Predicted DLCO/VA% correlated with HbA1c levels
Mohamad et al. 2011 [42]	Observational, case-control	60 (6–16 ys)	<FVC, <FEV1, <FEV1/FVC, <PEF	-	-	Subgroup analysis: patient with good vs. poor metabolic control (mean HbA1c < 8% vs. ≥8%)	EC and FEV1 were significantly lower in children with poor glycemic control No correlation with disease duration
Al-Saadi et al. 2011 [43]	Observational without control group	52 (8–14 ys)	<FVC, <PEF, <MMEF	-	-	-	NA
Scaramuzza et al. 2012 [20]	Observational, case-control	42, (10–20 ys)	<FVC, <FEV1	<TLC	<	<DLCO Vc	<DLCO in presence of autonomic neuropathy at neurovegetative test (index alfa) No correlation between pulmonary function test and DLCO and HbA1c, microalbuminuria, BMI, disease duration
Pieniawska et al. 2012 [44] *	Observational, case-control	35 (age range NA)	<VC >FEV1/FVC <PEF	-	-	-	No correlation between spirometric parameters and disease duration and HbA1c <VC related to high HbA1c
Martin-Frias et al. 2015 [21]	Observational, case-control	100 (8–18 ys)	< FVC, <FEV1 >FEV1/FVC	>TLC e RV >RV/TLC (<Raw)	<DLCO <VA >DLCO/AV	Results corrected for pubertal stage Subgroup analysis: patient with good vs. poor metabolic control (mean Hba1c < 7.5 vs. ≥7.5%)	No differences in pulmonary function based on duration of disease or metabolic control <FEV1/FVC in poor metabolic control subgroup
Anik et al. 2020 [45]	Observational, case-control	51 (3–15 ys)	<FEF 75 <FEF 25–75	(>Raw)	-	-	Positive: Metabolic control at the time of the test (HbA1c), disease duration

AV: Alveolar Volume; DLCO: Diffusing Lung Capacity of carbon monoxide; FEF: Forced Expiratory Flow; FEV1: Forced Expiratory Volume in 1 sec; FVC: Forced Vital Capacity; MMEF: Maximal Mid-Expiratory Flow; NA: not available; PEF: Peak Expiratory Flow; RV: Residual Volume; Vc: capillary blood volume; TLC: Total Lung Capacity; Ys: years; and -: not investigated; * article in Polish.

**Table 2:** Studies about T1D and asthma in pediatric age.

	Study Design	No. of T1D Patients (Age, ys)	Presence of Atopy	Allergic Test	Allergic Diseases	Asthma	Other Findings
Tosca et al. 2009 [60]	Observational Case-control	112 (7.8–16.9)	Sensitization to aeroallergenes similar to control group	SPT	No difference in the proportion of individuals with rhinits	No difference in the proportion of individuals with asthma	No T1D patients with “actual asthma” vs. 5, 8% in the control group (*p* = 0.009)
Jasser-Nitsche et al. 2017 [61]	Observational, Case-control	104 (11.4 ± 4.4)	Allergen-specific sensitization similar to control group	RAST	No difference in the proportion of individuals with symptoms		
Seiskari et al. 2018 [62]	Observational, Case-control	147 in Finland (NA); 132 in Karelian	No significant difference in allergen specific sensitization in Finland, more frequent sensitization in Karelian patients	Total serum IgE RAST (birch, cat, egg albumin)	Karelian T1D patients reported more frequent allergic diseases	Karelian T1D patients reported more frequent asthma	
Black et al. 2011 [63]	Observational, without control group	1683 (3–21)				Asthma was present in 10.0% of T1D youth	Youth with asthma had poor glycemic control
Klamt et al. 2015 [64]	Observational, Case-control	94 (12.12 ± 4.55)	No significant difference in allergen specific sensitization	Total serum IgE RAST	T1D patients have more risk for allergic symptoms	No difference in personal history of asthma	
Hsiao et al. 2015 [65]	Observational, Case-control	3545 (11.30 ± 5.04)				Incidence of asthma higher in T1D patients	HR of asthma higher in T1D patients hospitalized more than twice for diabetes.
Villa-Nova et al. 2015 [66]	Observational, without control group	96 (4–18)	Positive SPT in 46.9% of T1D patients	SPT	Higher prevalence of allergic symptoms: rhinitis 52.1%, atopic eczema 9.4%	Prevalence of asthma: 59.1%	
Hortenuber et al. 2004 [67]	Prospective observational study	51,926 patients (<20)				1755 patients (3, 4%) had the diagnosis of asthma	In T1D patients with asthma: Higher insulin dosage Lower height-SDS Higher BMI-SDS
Metsala et al. 2018 [68]	Observational, Case-control	8939 (5–14)		-		602 with T1D and asthma	Previous diagnosis of asthma increased the risk of T1D; previous diagnosis of T1D decreased the risk of asthma

BMI: body mass index; NA: not available; SDS: standard deviation score; SPT: skin prick tests; and Ys: years.
